# Serum fibroblast growth factor 21 is a novel biomarker of cachexia in chronic liver disease

**DOI:** 10.3389/fnut.2026.1730695

**Published:** 2026-02-18

**Authors:** Takatsugu Tanaka, Goki Suda, Masatsugu Ohara, Osamu Maehara, Tomoka Yoda, Qingjie Fu, Zijian Yang, Naohiro Yasuura, Akimitsu Meno, Takashi Sasaki, Risako Kohya, Takashi Kitagataya, Naoki Kawagishi, Masato Nakai, Takuya Sho, Shunsuke Ohnishi, Naoya Sakamoto

**Affiliations:** 1 Department of Gastroenterology and Hepatology, Graduate School of Medicine, Hokkaido University, Sapporo, Japan; 2 Laboratory of Molecular and Cellular Medicine, Faculty of Pharmaceutical Sciences, Hokkaido University, Sapporo, Japan

**Keywords:** biomarker, cachexia, chronic liver disease, fibroblast growth factor, liver cirrhosis

## Abstract

**Background:**

Cachexia is associated with poor prognosis in chronic liver disease (CLD), yet robust predictors remain poorly defined. This study examined clinical factors and serum biomarkers associated with cachexia in patients with CLD.

**Methods:**

We analyzed 356 of 526 patients with CLD who had complete cachexia assessment and available stored serum samples. In a discovery cohort (*n* = 240; Aug 2014–Jun 2023), serum fibroblast growth factor 21 (FGF21), interleukin-6 (IL-6), and tumor necrosis factor-*α* (TNF-α) were measured. Multivariable logistic regression and receiver operating characteristic analyses were used to identify independent predictors and optimal cutoff values. Findings were subsequently evaluated in an independent validation cohort (*n* = 116; Jul 2023–May 2025).

**Results:**

Median age was 68 years (range 19–90), 65.8% of participants were male, and 24.6% had cachexia, which independently predicted worse overall survival (hazard ratio 1.64; 95% CI 1.03–2.62; *p* = 0.038). Patients with cachexia had higher serum FGF21 concentrations than those without cachexia (median, 292 vs. 177 pg./mL; *p* = 0.002), whereas IL-6 and TNF-*α* levels did not differ significantly between groups. FGF21 was the only biomarker independently associated with cachexia (odds ratio, 1.71; 95% CI, 1.10–2.66; *p* = 0.016). Advanced Child–Pugh class and platelet count were identified as additional independent clinical predictors.

**Conclusion:**

Serum FGF21 independently predicts cachexia in CLD and may facilitate earlier identification of at-risk patients, enabling timely intervention to improve clinical outcomes.

## 1 Introduction

Cachexia is a systemic metabolic derangement characterized by the loss of body proteins, muscle atrophy, and anorexia, and is commonly associated with chronic illnesses, such as cancer, chronic heart failure, chronic obstructive pulmonary disease, chronic kidney disease, and chronic liver disease (CLD) (1, 2). Unlike simple starvation, cachexia involves a pronounced inflammatory response and metabolic alterations, leading to a disproportionate loss of skeletal muscle relative to fat. The syndrome severely impacts physical function, quality of life, and survival, making it a critical concern in the management of chronic diseases (3, 4).

In the context of liver disease, cachexia is frequently observed in patients with liver cirrhosis (LC) and hepatocellular carcinoma (HCC), both of which are associated with systemic inflammation, altered nutrient metabolism, and hormonal imbalances (5, 6). In these patients, cachexia is an independent predictor of poor prognosis, contributing to worsened functional status, reduced treatment tolerance, and higher mortality (7–11). The pathophysiology of cachexia in liver disease involves complex interactions among tumor-related factors, chronic inflammation, and hepatic dysfunction, all of which exacerbate progressive muscle wasting and metabolic disturbances.

Despite the clear association between cachexia and negative clinical outcomes in patients with LC and HCC (6, 8, 11), the specific factors that predict cachexia development remain poorly understood. Early identification of at-risk patients could lead to timely interventions aimed at preserving muscle mass and improving the overall prognosis.

In recent years, increasing attention has been paid to the identification of biomarkers for cachexia diagnosis and prognosis. In cancer-associated cachexia, numerous studies have investigated biomarkers such as serum albumin, C-reactive protein (CRP), tumor necrosis factor-alpha (TNF-*α*), and interleukin-6 (IL-6), among others (4, 12, 13). Fibroblast growth factor 21 (FGF21) is also a biomarker of interest. FGF21 is an endocrine metabolic hormone produced predominantly by the liver in response to nutritional and cellular stressors, such as fasting and endoplasmic reticulum stress (14). Its endocrine activity depends on binding to a fibroblast growth factor receptor (FGFR)–*β*-Klotho coreceptor complex, which is enriched in key metabolic target tissues, including adipose tissue and specific brain regions. Through this liver-to-periphery and liver-to-brain signaling axis, FGF21 can modulate appetite and energy expenditure, which are core features of cachexia (15, 16). Importantly, circulating FGF21 undergoes proteolytic cleavage at the C-terminus; therefore, assay design determines whether measured “FGF21” reflects intact bioactive hormone or a broader pool of immunoreactive fragments (17).

FGF21 is a crucial metabolic regulator (18) that plays a critical role in maintaining the energy balance and modulating carbohydrate and fat metabolism through paracrine and endocrine mechanisms (18). In several malignancies (19, 20), including HCC (21), serum FGF21 levels are significantly elevated. Its high serum levels may serve as a potential biomarker of poor prognosis in patients initially diagnosed with HCC (21). However, the underlying mechanisms remain unclear. To the best of our knowledge, there are no reports on whether high serum FGF21 levels predict cachexia in CLD.

Thus, in this study, we aimed to examine the potential predictive factors for cachexia in patients with LC and HCC. The findings of this study could help improve clinical management strategies for the diseases and improve patient outcomes.

## 2 Materials and methods

### 2.1 Patients and study design

**Figure 1:**
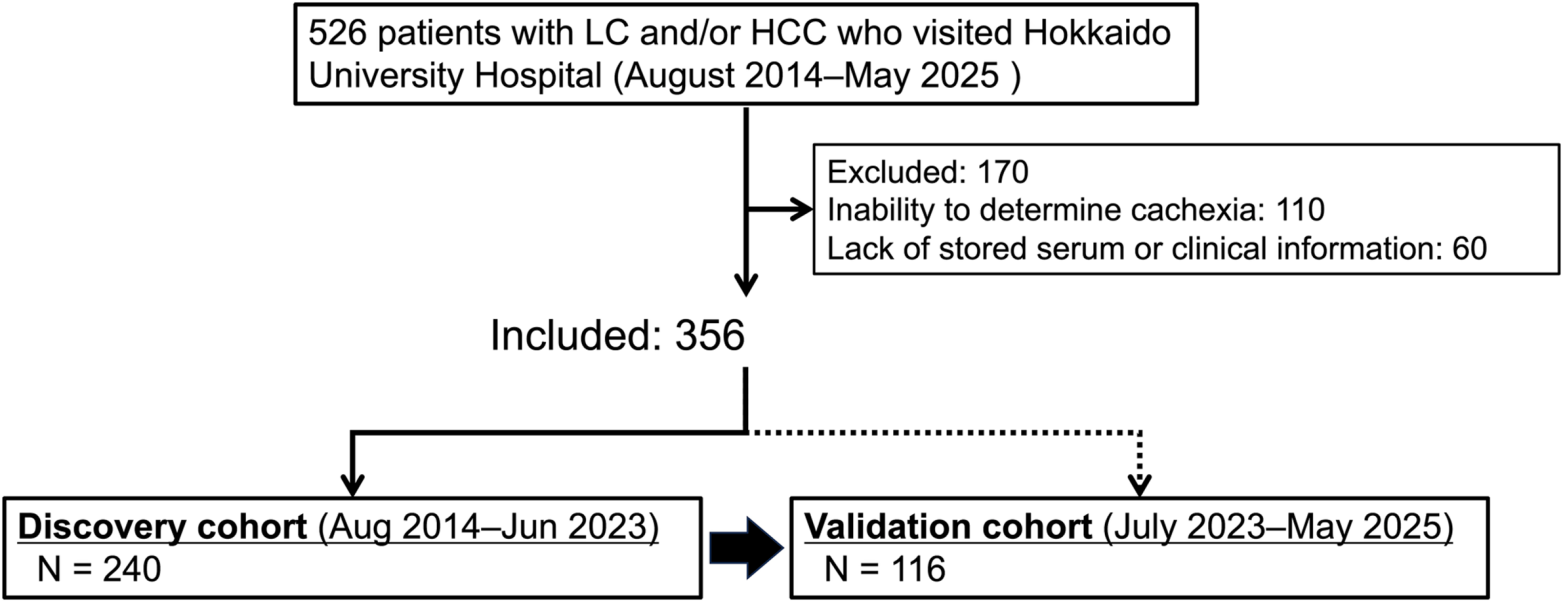
Flowchart of the study design and patient selection process. LC, liver cirrhosis; HCC, hepatocellular carcinoma.

In this retrospective study, we screened patients with LC and/or HCC who visited Hokkaido University Hospital between August 2014 and May 2025 (Figure 1). Patients were included if they had data on handgrip strength, comprehensive clinical information for analysis, and stored serum samples suitable for FGF21 measurement. Patients were excluded if they lacked data on handgrip strength, did not have appropriate clinical information, or did not have stored serum samples usable for measuring biomarkers, including FGF21, TNF-*α*, and IL-6. Among 526 patients screened, 356 were enrolled; of these, 240 patients enrolled between August 2014 and June 2023 were assigned to the discovery cohort, and the remaining 116 patients enrolled between July 2023 and May 2025 were assigned to the validation cohort.

The study collected patients’ data on sex, age, weight, body mass index (BMI), handgrip strength, presence of appetite loss, history of diabetes mellitus, etiology of liver disease, presence of LC, liver function (based on Child–Pugh class), presence of HCC, and date of death or last follow-up. The prevalence and prognosis of cachexia in patients with LC and/or HCC were assessed. The patients were divided into groups according to the presence or absence of cachexia, and the predictors of cachexia were investigated.

The study adhered to the ethical principles outlined in the Declaration of Helsinki. The Hokkaido University Hospital Ethics Committee approved the study protocol (approval numbers: 017-0521, 020-0267, 022-0052, and 023-0060). Participation in the study required written informed consent. Furthermore, in situations where formal written informed consent could not be secured, the Ethics Committee authorized the enrollment of patients who had previously granted broad permission for the use of their clinical specimens and data, provided that they had not expressly declined participation in this investigation.

### 2.2 Evaluation of serum biomarker levels

The baseline levels of the candidate serum biomarkers, including FGF21, TNF-*α*, and IL-6, were evaluated using commercial enzyme-linked immunosorbent assays according to the manufacturer’s protocols (FGF21 [Catalog #DF2100], TNF-α, and IL-6: R&D Systems, Minneapolis, MN, United States).

### 2.3 Definition of cachexia

Cachexia was defined based on a consensus report by the Asian Working Group for Cachexia (1). The diagnosis required the presence of an underlying chronic disease along with at least one of the following: (a) unintentional weight loss of ≥2% over 3–6 months, or (b) BMI < 21 kg/m^2^ and at least one of the following: (i) reduced handgrip strength (men <28 kg, women <18 kg), (ii) appetite loss, or (iii) elevated CRP level (>0.5 mg/dL).

### 2.4 Evaluation of serum FGF21 cutoff values

Receiver operating characteristic (ROC) curve analysis was performed using serum FGF21 levels as the independent variable and cachexia as the outcome in the discovery cohort. The optimal cutoff value for serum FGF21 levels was determined using Youden’s index. Based on the calculated baseline cutoff value, the cohort was classified into high-FGF21 and non-high-FGF21 categories. Using the established cutoff value, we performed a comparative analysis of cachexia prevalence in the discovery and validation cohorts. We also evaluated the effect of cachexia on the prognosis of patients with CLD.

### 2.5 Statistical analysis

For the analysis of categorical data, the Chi-squared or Fisher’s exact test was used, as appropriate. Continuous variables were evaluated using the Mann–Whitney U test. Survival curves were constructed using the Kaplan–Meier method, and differences between these curves were assessed using the log-rank test. Cox proportional hazards regression models were used to identify independent prognostic factors and adjust for confounding variables correlated with the observed endpoint. Multivariable logistic regression analysis was used to identify independent predictors of cachexia, including variables that met the predefined screening criterion (*p* < 0.10) in univariable analyses. ROC analyses were performed, and the area under the ROC curve (AUROC) was estimated with 95% confidence intervals. When multiple thresholds demonstrated comparable performance near the maximum Youden index (J = sensitivity + specificity − 1), a cutoff within this plateau was selected to balance sensitivity and specificity and enhance clinical interpretability. Sensitivity and specificity at the selected cutoff are reported. Statistical significance was set at *p* < 0.05. All statistical analyses were performed using Prism 9.41 (GraphPad Software, La Jolla, CA, United States).

## 3 Results

### 3.1 Patient characteristics

A total of 240 and 116 patients were included in the discovery and validation cohorts, respectively (Figure 1).

The baseline patient characteristics are shown in Table 1. Two hundred twelve patients (88.3%) had LC, and 135 (56.3%) had HCC. Serum FGF21, TNF-*α*, and IL-6 levels were 191.23 (range: 17.44–5,282.80) pg./mL, 46.37 (range: 0.06–619.53) pg./mL, and 3.17 (range: 0.35–490.29) pg./mL, respectively.

**Table 1:** Baseline characteristics of patients with chronic liver disease, stratified by cachexia status.

Sample characteristics	Overall (*n* = 240)	No cachexia (*n* = 181)	Cachexia (*n* = 59)	*p*-value
Age, years	68 (63–75)	68 (63–73)	72 (65–76)	0.042
Sex, male/female	158/82	124/57	34/25	0.155
BMI, kg/m^2^	24.54 (22.18–26.91)	24.86 (22.53–27.10)	22.34 (19.84–26.54)	0.003
Decreased grip strength, No/Yes	148/92	129/52	19/40	< 0.001
Body fat percentage, %	27.50 (23.05–33.75)	28.50 (23.80–33.90)	25.75 (20.53–33.38)	0.168
Appetite loss, no/yes	202/38	166/15	36/23	< 0.001
Diabetes mellitus, no/yes	162/78	128/53	34/25	0.078
Intake of BCAA supplements, no/yes	154/86	115/66	39/20	0.757
Etiology, %				0.293
Viral	119 (49.6%)	95	24	
Alcohol	49 (20.4%)	37	12	
MASH	44 (18.3%)	29	15	
Other	28 (11.7%)	20	8	
Child–Pugh class, %				0.183
A	153 (63.7%)	121	32	
B	75 (31.2%)	52	23	
C	12 (5.1%)	8	4	
HCC, No/Yes	105/135	84/97	21/38	0.174
Biochemical analysis				
Platelet, ×10^4^/μL	11.30 (8.20–16.30)	11.10 (7.60–15.50)	13.30 (9.80–17.90)	0.020
AST, IU/L	34.00 (25.00–50.00)	32.00 (25.00–48.00)	38.00 (29.00–59.00)	0.012
ALT, IU/L	25.00 (18.00–35.00)	24.00 (17.00–35.00)	26.00 (21.50–41.50)	0.052
Serum albumin, g/dL	3.90 (3.48–4.23)	3.90 (3.50–4.30)	3.80 (3.25–4.20)	0.110
CRP, mg/dL	0.09 (0.03–0.40)	0.08 (0.03–0.23)	0.45 (0.05–1.05)	< 0.001
Survival time from enrollment, days	1,556 (950–2,590)	1793 (1018–2,703)	1,200 (572–1957)	0.001

Data are presented as number (%) or median (interquartile range). ALT, alanine aminotransferase; AST, aspartate aminotransferase; BCAA, branched-chain amino acids; BCLC, Barcelona Clinic Liver Cancer; BMI, body mass index; CRP, C-reactive protein; HCC, hepatocellular carcinoma; MASH, metabolic dysfunction-associated steatohepatitis.

### 3.2 Prevalence of cachexia

Of 240 patients, 59 (24.6%) were diagnosed with cachexia (Table 1). For each component of cachexia, the prevalence of weight loss, low BMI, appetite loss, decreased grip strength, and elevated CRP (> 0.5 mg/dL) was 22.9, 14.6, 15.8, 36.3, and 21.3%, respectively.

### 3.3 Prognostic impact of cachexia in CLD

**Figure 2:**
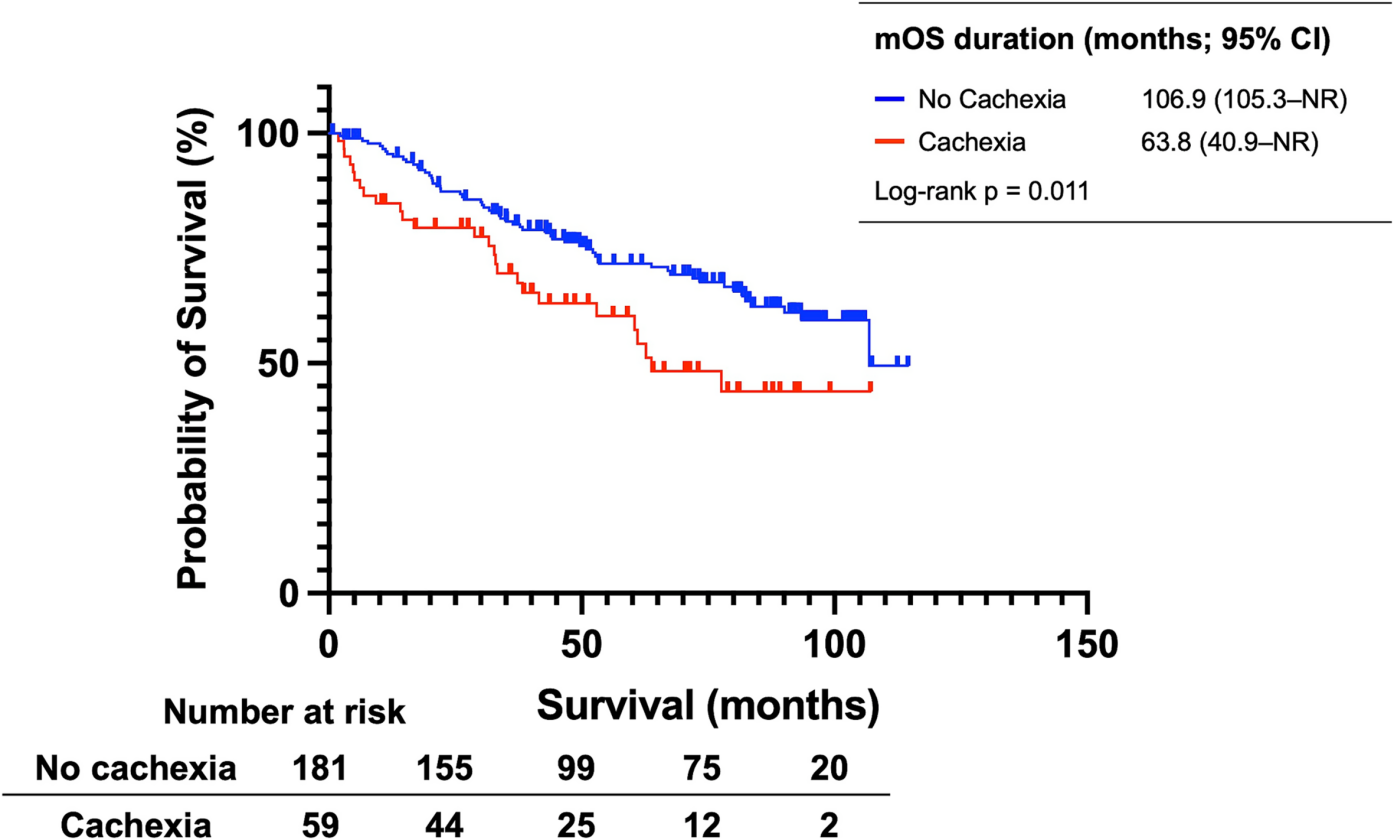
Kaplan–Meier estimates of overall survival in the entire discovery cohort, stratified by the presence or absence of cachexia. mOS, median overall survival; CI, confidence interval; NR, not reached.

A comparison of overall survival (OS) in patients with and without cachexia is shown in Figure 2. Patients with cachexia had significantly shorter median OS than those without (63.8 months [95% confidence interval (CI): 40.9–not reached (NR)] vs. 106.9 months [95% CI: 105.3–NR], *p* = 0.011).

Subsequent univariable and multivariable Cox regression analyses for OS identified HCC (hazard ratio [HR]: 3.325 [95% CI: 2.021–5.470], *p* < 0.001) and cachexia (HR: 1.638 [95% CI: 1.027–2.610], *p* = 0.038) as independent risk factors (Table 2).

**Table 2:** Cox proportional hazards regression analysis of overall survival.

	Univariable analysis		Multivariable analysis	
	HR (95% CI)	*p*-value	HR (95% CI)	*p*-value
Age, per year	1.018 (0.996–1.040)	0.109		
Sex, male vs. female	0.746 (0.467–1.191)	0.219		
Etiology				
Viral	1 (reference)			
Alcohol	1.152 (0.671–1.977)	0.608		
MASH	1.240 (0.698–2.205)	0.463		
Other	0.759 (0.341–1.686)	0.498		
Child–Pugh class. A vs. B/C	1.429 (0.925–2.207)	0.108		
HCC (yes vs. no)	3.439 (2.093–5.650)	< 0.001	3.325 (2.021–5.470)	< 0.001
Cachexia (yes vs. no)	1.810 (1.137–2.882)	0.012	1.638 (1.027–2.610)	0.038

CI, confidence interval; HCC, hepatocellular carcinoma; HR, hazard ratio; MASH, metabolic dysfunction-associated steatohepatitis.

### 3.4 Predictive factors for cachexia in patients with CLD

**Figure 3:**
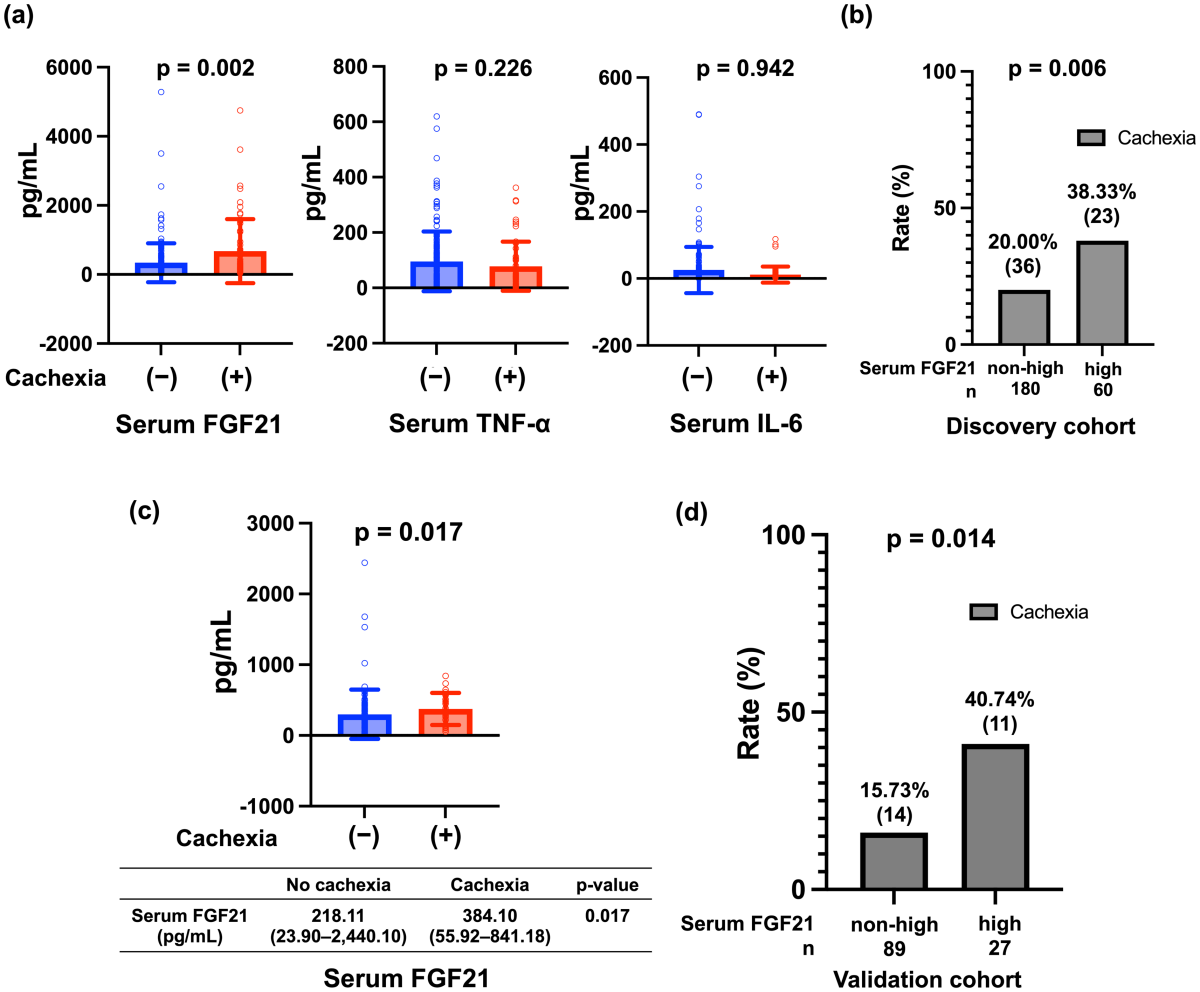
**(a)** Comparison of serum FGF21, TNF-*α*, and IL-6 levels between patients with and without cachexia. **(b)** Comparison of the proportion of cachexia between patients with high and low serum FGF21 levels in the discovery cohort. **(c)** Comparison of serum FGF21 levels between patients with and without cachexia in the validation cohort. **(d)** Comparison of the proportion of cachexia cases between patients with high and low FGF21 levels in the validation cohort. FGF, Fibroblast Growth Factor; TNF, Tumor Necrosis Factor; IL, Interleukin.

A comparison of patient characteristics with and without cachexia is shown in Table 1. The median age was higher in patients with cachexia than in those without (*p* = 0.042). Patients with cachexia had a significantly lower BMI (*p* = 0.003) and were significantly more likely to have appetite loss (*p* < 0.001) and decreased grip strength (*p* < 0.001) than those without. No significant differences were found in etiology, presence of LC, Child–Pugh class, or HCC. CRP levels were significantly higher, and the survival period from entry was significantly shorter in the cachexia group than in the non-cachexia group (*p* = 0.001). As shown in Figure 3a, serum TNF-*α* and IL-6 levels did not differ significantly between groups with and without cachexia. Median serum FGF21 levels were significantly higher in the cachexia group than in the non-cachexia group (292.02 vs. 176.93 pg./mL, *p* = 0.002).

Based on the above results, a univariable logistic regression analysis was performed to identify factors contributing to the diagnosis of cachexia. The etiology (metabolic dysfunction-associated steatohepatitis), Child–Pugh class (A vs. B/C), platelet count, and serum FGF21 levels were associated with cachexia (*p* < 0.10) in univariable analysis and met the prespecified screening threshold (Table 3). These variables were included in the multivariable logistic regression model, which showed that Child–Pugh class (A vs. B/C), platelet count, and serum FGF21 levels remained significant independent predictors of cachexia; serum FGF21 levels had an odds ratio of 1.710 (95% CI: 1.110–2.650; *p* = 0.016) (Table 3).

**Table 3:** Multivariable logistic regression analysis of factors associated with cachexia.

	Univariable analysis		Multivariable analysis	
	OR (95% CI)	*p*-value	OR (95% CI)	*p*-value
Age, per year	1.020 (0.995–1.060)	0.100		
Sex, male vs. female	1.600 (0.874–2.930)	0.128		
Etiology				
Viral	1 (Reference)		1 (Reference)	
Alcohol	1.280 (0.583–2.830)	0.536		
MASH	2.050 (0.950–4.410)	0.067	1.610 (0.723–3.580)	0.244
Other	1.580 (0.622–4.030)	0.335		
Child–Pugh class, A vs. B/C	1.700 (0.935–3.100)	0.082	1.940 (1.020–3.680)	0.044
HCC (yes vs. no)	1.570 (0.853–2.880)	0.147		
Platelet, per ×10^4^/μL	1.040 (1.010–1.080)	0.018	1.040 (1.000–1.080)	0.030
AST, IU/L	1.000 (0.997–1.010)	0.264		
ALT, IU/L	1.000 (0.996–1.010)	0.523		
Serum FGF21, per ng/mL	1.890 (1.220–2.930)	0.004	1.710 (1.110–2.650)	0.016

ALT, alanine aminotransferase; AST, aspartate aminotransferase; CI, confidence interval; FGF, fibroblast growth factor; HCC, hepatocellular carcinoma; MASH, metabolic dysfunction-associated steatohepatitis; OR, odds ratio.

Based on the ROC curve analysis of serum FGF21 levels in 240 patients from the discovery cohort, a cutoff value of 426 pg./mL was identified to predict cachexia among patients with CLD, yielding a sensitivity of 39.0%, a specificity of 79.6%, a positive predictive value of 0.383, and a negative predictive value of 0.800 (Supplementary Figure S1). Patients with serum FGF21 levels ≥ 426 pg./mL were defined as having high FGF21 levels. Cachexia prevalence was significantly higher in the high-FGF21 group than in the non-high-FGF21 group (38.33% vs. 20.00%; *p* = 0.006; Figure 3b).

### 3.5 Independent validation of serum FGF21 levels as a predictor of cachexia in patients with CLD

The results were validated in an independent cohort of 116 patients with LC and/or HCC (Figure 1; Supplementary Table 2). Baseline characteristics of the cohort and comparisons between patients with and without cachexia are summarized in the Supplementary Table S1. A total of 25 patients (24.6%) had cachexia.

Median serum FGF21 levels were significantly higher in the cachexia group than in the non-cachexia group (384.10 [range: 23.90–2,440.10] pg./mL vs. 218.11 [range: 55.92–841.18] pg./mL, *p* = 0.017; Figure 3c).

The findings from the discovery cohort were reproduced in the validation cohort. Using a cutoff value of 426 pg./mL, the high-FGF21 group exhibited a significantly higher prevalence of cachexia than the non-high-FGF21 group (40.74% vs. 15.73%; *p* = 0.014; Figure 3d).

## 4 Discussion

This study demonstrated for the first time that serum FGF21 levels are a robust diagnostic predictor of cachexia in patients with CLD. Of the 240 patients in the discovery cohort, 24.6% were diagnosed with cachexia, and serum FGF21 levels were significantly higher in patients with cachexia than in those without. Multivariable logistic regression analysis further demonstrated that serum FGF21 levels were an independent factor associated with cachexia. These findings were validated through an independent analysis of a separate cohort of 116 patients. Moreover, patients classified into the high-FGF21 group based on the cutoff value derived from the discovery cohort showed a significantly higher prevalence of cachexia than those in the non-high-FGF21 group in both the discovery and validation cohorts. Previous reports have noted elevated FGF21 levels in older patients with cachexia (22), and overall research on FGF21 as a biomarker remains limited. Furthermore, to the best of our knowledge, no study has specifically investigated FGF21 as a biomarker in patients with CLD. The present study demonstrates that serum FGF21 levels may serve as a predictive marker for cachexia in patients with HCC and those with CLD.

Although a causal relationship between cachexia and elevated serum FGF21 levels remains unclear, several mechanisms have been proposed. Elevated FGF21 levels can suppress appetite via endocrine signaling in central nervous system networks (23, 24). FGF21 activates brown adipose tissue in both autocrine and paracrine manners, promoting lipolysis and thermogenesis by browning white adipose tissue (25), thereby increasing energy expenditure and leading to subsequent weight loss (26, 27). Moreover, FGF21 upregulation in response to fasting, endoplasmic reticulum stress, or metabolic disorders has been implicated in muscle atrophy and decreased muscle strength (28), contributing to further body weight reduction. Clinical studies support these results, reporting associations between elevated serum FGF21 levels and appetite or weight loss (29), as well as significantly elevated serum FGF21 levels in patients with muscle weakness (30). Given that weight loss, appetite loss, and muscle weakness are key diagnostic criteria for cachexia, these mechanisms may be associated with the observed elevation in serum FGF21 levels in patients with cachexia. However, further research is required to validate and expand upon these findings.

Circulating FGF21 undergoes proteolytic processing *in vivo*, and C-terminal truncation reduces biological activity by impairing *β*-Klotho binding (31). Notably, fibroblast activation protein (FAP) can cleave human FGF21, generating an inactive C-terminally truncated form (32). Accordingly, measured “FGF21” concentrations may depend on assay design and the specific molecular species detected; thus, results obtained using our ELISA should be interpreted as reflecting circulating immunoreactive FGF21 rather than exclusively intact (biologically active) hormone. Recent studies have employed alternative commercial ELISAs to quantify the active moiety of FGF21 (33). Future studies should prospectively collect sufficient sample aliquots to enable separate quantification of intact (active) and truncated (inactive) FGF21.

In the discovery cohort of the present study, Child–Pugh class and platelet count were identified as independent predictors of cachexia. Previous studies have demonstrated that the prevalence of cachexia increases significantly with the progression of Child–Pugh class (11), which is consistent with our findings. However, it remains unclear whether impaired hepatic functional reserve contributes to the development of cachexia or whether cachexia itself leads to hepatic deterioration. Further investigation is warranted to clarify this relationship. Platelets induce the release of inflammatory mediators from immune cells, contributing to the inflammatory response characteristics of cachexia (34). In our study, CRP levels were significantly elevated in patients with cachexia, supporting the hypothesis that systemic inflammation plays a key role. This may explain why the platelet count was identified as a predictive factor for cachexia in the multivariable analysis.

Given that the prevalence and prognosis of cachexia in CLD have not been fully elucidated, our study provides additional insights into these aspects by presenting relevant data on the prevalence and prognostic impact of cachexia in CLD. In the present study, the prevalence of cachexia in patients with CLD was 24.6%. Patients with cachexia had a significantly shorter OS than those without cachexia. Furthermore, multivariable Cox regression analysis confirmed that cachexia is an independent factor associated with OS. In the present study, the observed prevalence was comparable to that in previous reports, including a prevalence of 23.7% among patients with HCC reported by Rich et al. (6) and the findings of a scoping review by Unome et al. (10), which reported cancer-related and non-cancer-related cachexia prevalence rates among Asian populations ranging from 6.2 to 93% and 3.4 to 66.2%, respectively. This study also demonstrates that cachexia is an independent predictor of poor prognosis in patients with CLD. Previous studies investigating prognosis in patients with HCC and LC have also identified cachexia as an independent adverse prognostic factor (6, 8, 11). Therefore, our findings are consistent with those of previous studies.

This study demonstrated that serum FGF21 levels may be a promising diagnostic predictor of cachexia in patients with CLD. Given that cachexia is associated with a poor prognosis, early detection and intervention are essential for improving clinical outcomes. As serum FGF21 levels can be measured using a simple blood test, they may serve as a useful tool for the early identification of cachexia, which is otherwise diagnosed using complex clinical criteria. Although serum FGF21 was strongly associated with cachexia in our cohort, this association does not establish causality. Elevated FGF21 may contribute to appetite suppression and increased energy expenditure, thereby exacerbating weight loss; however, reverse causality is also plausible, as cachexia-related undernutrition and systemic or hepatic stress may upregulate FGF21. Accordingly, our findings should be interpreted as demonstrating a clinically relevant association. Future studies incorporating longitudinal sampling and experimental approaches are required to clarify the directionality and underlying mechanisms of this relationship.

This study has limitations, including its retrospective design and the fact that it was conducted at a single center with a relatively small sample size. In particular, the number of patients for whom additional data on handgrip strength were available was limited. Dietary intake and overall nutritional patterns were not comprehensively assessed in this study; therefore, we could not fully account for the potential influence of energy intake on circulating FGF21 concentrations. Therefore, a large-scale prospective study is needed to validate these findings.

In conclusion, this study is the first to demonstrate the potential utility of serum FGF21 levels as a diagnostic predictor of cachexia in patients with CLD. These findings suggest that serum FGF21 may be a valuable tool for early diagnosis and intervention in cachexia, potentially improving clinical outcomes in this patient population.

## Data Availability

The datasets presented in this article are not readily available because the datasets generated during this study are not publicly accessible due to ethical restrictions and the need to protect patient privacy. Requests to access the datasets should be directed to GS, gsudgast@pop.med.hokudai.ac.jp.
