# Supplementary material for: Serum fibroblast growth factor 21 is a novel biomarker of cachexia in chronic liver disease
Source: Front Nutr. 2026 Feb 18;13:1730695. doi: 10.3389/fnut.2026.1730695 (PMC12956663; doi:10.3389/fnut.2026.1730695)
Supplement: Supplementary file 1 [file Table_1.docx]

Supplementary Material

# Supplementary Figures and Tables

## Supplementary Tables

## Supplementary Table 1. Baseline patient characteristics and comparison between patients with and without cachexia in the validation cohort.

| Sample characteristics | Overall | No cachexia | Cachexia | *p*-value |
| --- | --- | --- | --- | --- |
|  | (n = 116) | (n = 91) | (n = 25) |  |
| Age, years | 71 (59–77) | 69 (55–77) | 73 (66–77) | 0.263 |
| Sex, male/female | 77 / 39 | 61 / 30 | 16 / 9 | 0.813 |
| BMI, kg/m^2^ | 25.28 (22.90–28.67) | 25.97 (23.86–29.14) | 22.15 (20.61–26.61) | < 0.001 |
| Decreased grip strength, no/yes | 58 / 58 | 57 / 34 | 1 / 24 | < 0.001 |
| Appetite loss, no/yes | 113 / 3 | 89 / 2 | 24 / 1 | 0.521 |
| Diabetes mellitus, no/yes | 67 / 49 | 54 / 37 | 13 / 12 | 0.648 |
| Intake of BCAA supplements, no/yes | 83 / 33 | 66 / 25 | 17 / 8 | 0.628 |
| Etiology |  |  |  | 0.396 |
| Viral | 35 (30.2%) | 30 | 5 |  |
| Alcohol | 20 (17.2%) | 14 | 6 |  |
| MASH | 46 (39.7%) | 34 | 12 |  |
| Other | 15 (12.9%) | 13 | 2 |  |
| Child–Pugh class |  |  |  | 0.828 |
| A | 91 (78.4%) | 72 | 19 |  |
| B | 24 (20.7%) | 18 | 6 |  |
| C | 1 (0.9%) | 1 | 0 |  |
| HCC, no/yes | 75 / 41 | 62 / 29 | 13 / 12 | 0.160 |
| Biochemical analysis |  |  |  |  |
| Platelet, ×10^4^/μL | 13.60 (9.35–19.63) | 13.70 (9.05–19.4) | 13.60 (10.10–21.00) | 0.822 |
| AST, IU/L | 31.00 (22.00–40.25) | 30.00 (22.00–39.00) | 32.00 (23.00–46.00) | 0.323 |
| ALT, IU/L | 21.50 (16.00–34.00) | 22.00 (17.00–34.00) | 20.00 (15.00–52.00) | 0.817 |
| Serum albumin, g/dL | 3.90 (3.50–4.30) | 3.90 (3.60–4.28) | 3.70 (3.40–4.30) | 0.820 |
| CRP, mg/dL | 0.11 (0.04–0.40) | 0.08 (0.04–0.28) | 0.24 (0.05–0.75) | 0.089 |

Data are presented as number (%) or median (interquartile range). ALT, alanine aminotransferase; AST, aspartate aminotransferase; BCAA, branched-chain amino acids; BMI, body mass index; CRP, C-reactive protein; HCC, hepatocellular carcinoma; MASH, metabolic dysfunction-associated steatohepatitis.

## Supplementary Table 2. Comparison of baseline characteristics between the discovery and validation cohorts

| Sample characteristics | Discovery cohort | Validation cohort | *p*-value |
| --- | --- | --- | --- |
|  | (n = 240) | (n = 116) |  |
| Age, years | 68 (63–75) | 71 (59–77) | 0.495 |
| Sex, male/female | 158 / 82 | 77 / 39 | 1.000 |
| BMI, kg/m^2^ | 24.54 (22.18–26.91) | 25.48 (22.90–28.67) | 0.019 |
| Decreased grip strength, no/yes | 148 / 92 | 58 / 58 | 0.040 |
| Appetite loss, no/yes | 202 / 38 | 113 / 3 | < 0.001 |
| Cachexia, no/yes | 181 / 59 | 91 / 25 | 0.595 |
| Diabetes mellitus, no/yes | 162 / 78 | 67 / 49 | 0.078 |
| Intake of BCAA supplements, no/yes | 154 / 86 | 83 / 33 | 0.188 |
| Etiology |  |  | < 0.001 |
| Viral | 119 | 35 |  |
| Alcohol | 49 | 20 |  |
| MASH | 44 | 46 |  |
| Other | 28 | 15 |  |
| Child–Pugh class |  |  | 0.008 |
| A | 153 | 91 |  |
| B | 75 | 24 |  |
| C | 12 | 1 |  |
| HCC, No/Yes | 105 / 135 | 75 / 41 | < 0.001 |
| Biochemical analysis |  |  |  |
| Platelet, ×10^4^/μL | 11.30 (8.20–16.30) | 13.60 (9.35–19.63) | 0.010 |
| AST, IU/L | 34.00 (25.00–50.00) | 31.00 (22.00–40.25) | 0.011 |
| ALT, IU/L | 25.00 (18.00–35.00) | 21.50 (16.00–34.00) | 0.210 |
| Serum albumin, g/dL | 3.90 (3.48–4.23) | 3.90 (3.50–4.30) | 0.567 |
| CRP, mg/dL | 0.09 (0.03–0.40) | 0.11 (0.04–0.40) | 0.814 |

Data are presented as number (%) or median (interquartile range). ALT, alanine aminotransferase; AST, aspartate aminotransferase; BCAA, branched-chain amino acids; BMI, body mass index; CRP, C-reactive protein; HCC, hepatocellular carcinoma; MASH, metabolic dysfunction-associated steatohepatitis.

## Supplementary Figures

**
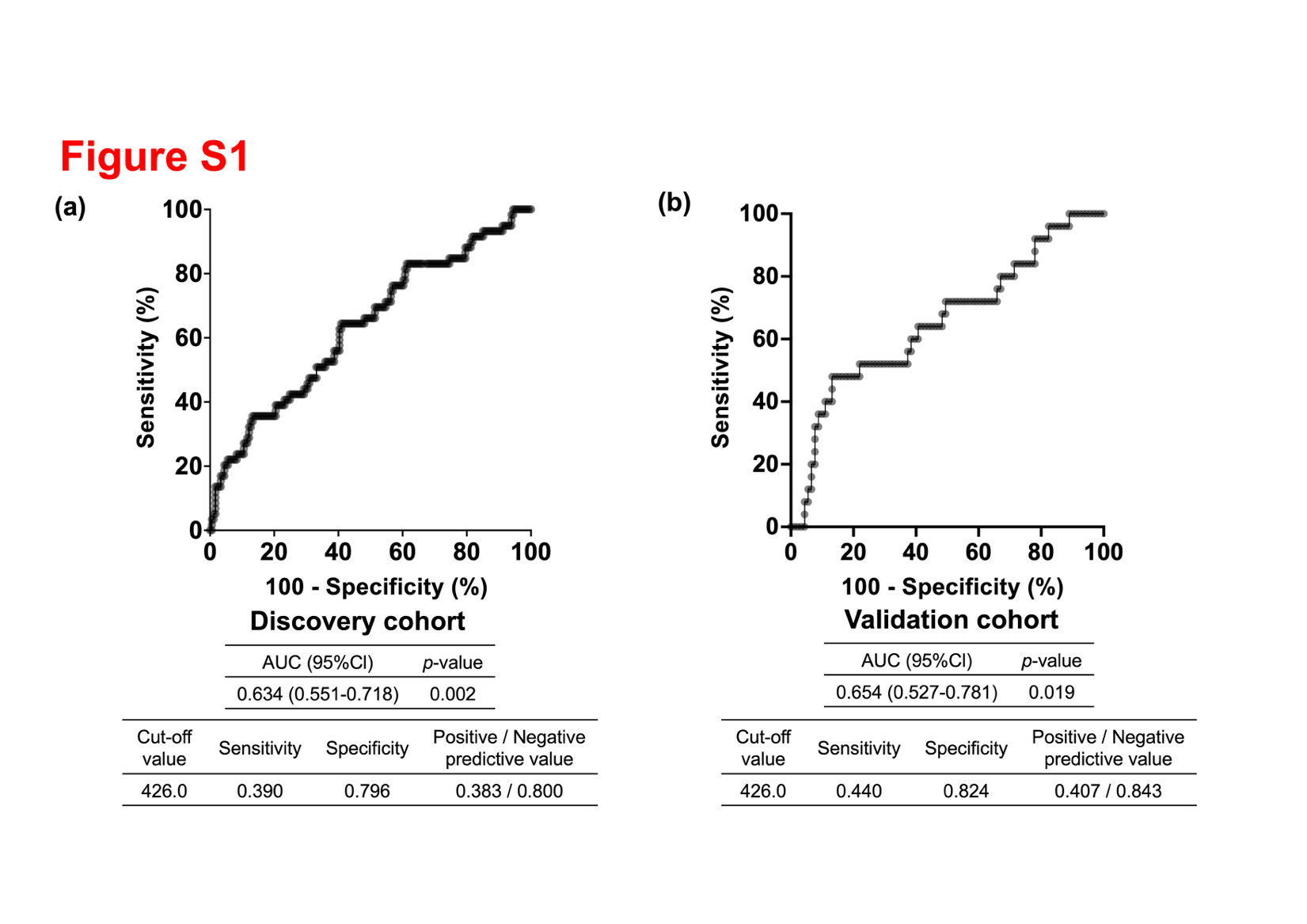
**

## Supplementary Figure S1. Receiver operating characteristic (ROC) curves of serum FGF21 for predicting cachexia. (a) Discovery cohort. The optimal cutoff value for serum FGF21 was 426 pg/mL, determined using Youden index, yielding a sensitivity of 39.0%, specificity of 79.6%, PPV of 38.3%, and NPV of 80.0%. (b) Validation cohort. Using the predefined cutoff value of 426 pg/mL, the sensitivity was 44.0%, specificity was 82.4%, PPV was 40.7%, and NPV was 84.3%. AUC, area under the receiver operating characteristic curve; ROC, receiver operating characteristic; FGF21, fibroblast growth factor 21; PPV, positive predictive value; NPV, negative predictive value.
